# The Effect of Casein Protein Prior to Sleep on Fat Metabolism in Obese Men

**DOI:** 10.3390/nu8080452

**Published:** 2016-07-27

**Authors:** Amber W. Kinsey, Stacy R. Cappadona, Lynn B. Panton, Brittany R. Allman, Robert J. Contreras, Robert C. Hickner, Michael J. Ormsbee

**Affiliations:** 1 Department of Nutrition Sciences, University of Alabama at Birmingham, Birmingham, AL 35294, USA; akinsey@uab.edu; 2 Nutrition Obesity Research Center, University of Alabama at Birmingham, Birmingham, AL 35294, USA; 3 Institute of Sports Sciences & Medicine, Department of Nutrition, Food and Exercise Sciences, Florida State University, Tallahassee, FL 32306, USA; src06e@my.fsu.edu (S.R.C.); lpanton@fsu.edu (L.B.P.); bra13@my.fsu.edu (B.R.A.); 4 Institute of Successful Longevity, Department of Nutrition, Food and Exercise Sciences, Florida State University, Tallahassee, FL 32306, USA; 5 Department of Psychology, Florida State University, Tallahassee, FL 32306, USA; rcontreras@fsu.edu; 6 Human Performance Laboratory, Departments of Kinesiology and Physiology and Center for Health Disparities, East Carolina University, Greenville, NC 27858, USA; hicknerr@ecu.edu; 7 Discipline of Biokinetics, Exercise and Leisure Sciences, School of Health Sciences, University of KwaZulu-Natal, Durban 4000, South Africa

**Keywords:** nighttime eating, sleep, obesity, microdialysis, appetite, casein

## Abstract

We have previously shown that ingesting protein at night before sleep is either beneficial or non-detrimental to metabolism, health, and body composition in obese women. However, the overnight protein-induced lipolytic actions and mechanism for improved metabolism and body composition have not been fully established. Therefore, in a crossover design, twelve obese men (age, 27.0 ± 2.2 years) were randomly assigned to ingest (within 30 min of sleep) casein protein (CAS, 120 kcal) or a non-nutritive placebo (PLA) before going to sleep. Markers of fat metabolism (lipolysis, substrate utilization, growth hormone), insulin, glucose, resting energy expenditure (REE), and appetite (questionnaire and ghrelin) were measured. During sleep and the next morning, interstitial glycerol from the subcutaneous abdominal adipose tissue (SCAAT) was measured using microdialysis. There were no differences in SCAAT glycerol (overnight: CAS, 177.4 ± 26.7; PLA, 183.8 ± 20.2 μmol/L; morning: CAS, 171.6 ± 19.1; PLA, 161.5 ± 18.6 μmol/L), substrate utilization, REE, or any blood markers between CAS and PLA. Desire to eat was greater for CAS compared to baseline (*p* = 0.03), but not different from PLA (baseline: 39 ± 6, CAS: 62 ± 8, PLA: 55 ± 5 mm). CAS consumption before sleep did not affect fat or glucose metabolism, REE, or suppress appetite in hyperinsulemic obese men. CAS may be consumed before sleep without impeding overnight or morning fat metabolism in young, obese men.

## 1. Introduction

Previous research indicates that high caloric meals [1,2,3] ingested late at night contribute to weight gain and impaired cardiometabolic health [4,5]. However, recent data on the health impact of nighttime eating suggests that our understanding of this practice may need modification based on the criteria used to identify late night eating (e.g., majority of daily intake solely at night, consumed during a specified time frame at night, includes dinner, or minutes before bedtime), as well as the content (percent carbohydrate vs. fat vs. protein) and quality (glycemic index) of calories consumed during this period [6].

Recent work [7,8,9,10,11,12,13] suggests that low-calorie (~150 kcals), protein-rich sources may be beneficial or non-detrimental to metabolism, health, and body composition when consumed as a snack ~30 min before going to sleep acutely (one-night) [7,8,9,11,12] and when combined with exercise for a longer duration (i.e., 4–12 weeks) [10,13]. In general, protein ingestion suppresses appetite, increases energy expenditure and muscle mass, and decreases body fat [14,15,16,17,18], encouraging support for ingesting protein-rich foods before going to sleep. Casein protein (CAS) has been designated as an optimal bedtime snack because it is slow to digest and augments muscle protein synthesis [19,20] making it ideal for pre-sleep ingestion. Indeed, data show that acute CAS intake (30–40 g per serving), compared to a non-nutritive placebo, is properly digested/absorbed and increases overnight muscle protein synthesis when provided before sleep [11,12]. We have previously demonstrated that casein before bed elevates next morning resting energy expenditure (REE) more than a non-nutritive placebo, but does not inhibit fat oxidation, as observed with other proteins and macronutrients relative to placebo, in normal-weight men [9]. This may suggest that casein before bed may not negatively impair fat metabolism relative to fasting and therefore may be appropriate to consume in close proximity to sleep. Studies in obese women have demonstrated that CAS ingestion before sleep can increase next morning satiety and, augment REE from baseline, however no differences were detected between casein and calorically-matched whey protein or carbohydrate ingestion [8]. Additionally, regardless of macronutrient type, acute elevations in insulin and insulin resistance have been observed the morning after pre-sleep caloric intake in obese women [8]. While the latter finding is not ideal for obese populations, it is important to note that a true non-nutritive placebo was absent and food intake throughout the day was not well controlled [8]. It is possible that the habitual diet during the day could have confounded the effects of nighttime macronutrient ingestion on next morning insulin and, hence, deserves further attention.

The potential mechanisms for improved metabolism and body composition have not been fully explored. To our knowledge, no studies have investigated mechanisms by which pre-sleep protein ingestion alters body fat (i.e., lipolysis) during sleep. Elucidating these mechanisms may be clinically relevant especially for obese individuals as eating the wrong foods at the wrong time (i.e., late at night before sleep) may contribute to increases in body fat and obesity [3,21]. Upper body subcutaneous fat depots are the predominate suppliers of circulating free fatty acids during the overnight postabsorptive period [22] and investigating changes in subcutaneous abdominal adipose tissue (SCAAT) lipolysis with microdialysis during sleep provides a novel opportunity to explore changes in regional fat metabolism that may ensue as a result of bedtime protein ingestion. Therefore, the purpose of this study was to examine the effect of CAS consumption before sleep on SCAAT lipolysis, whole-body substrate utilization, growth hormone, insulin, glucose, and REE compared to consumption of a non-nutritive placebo (PLA) in obese men. Based on our earlier work showing no differences in morning fat oxidation following bedtime CAS as compared to PLA ingestion in normal weight men [9], we hypothesized that CAS and PLA would yield similar changes in SCAAT lipolysis, whole-body substrate utilization (favoring fat oxidation), growth hormone, insulin and glucose in obese men. We also hypothesized that CAS prior to sleep will increase next morning REE more than PLA, as previously reported [8,9]. By measuring SCAAT lipolysis with microdialysis and whole-body fat oxidation, we examined the mechanisms by which CAS consumption before sleep may mediate changes in metabolism and body composition. In addition, we also decided to explore the effect of CAS before sleep on subjective feelings of appetite and explore potential mechanisms for these changes, if any, by measuring ghrelin, an appetite hormone. Others have shown that nutrient ingestion prior to sleep may suppress next morning appetite [8,12] but no studies have explored mediating mechanisms. Likewise, whether this holds true in obese men is unknown. It was hypothesized that CAS will decrease appetite to a significantly greater extent than PLA while its effect on ghrelin is exploratory.

## 2. Materials and Methods

### 2.1. Participants

Participants were recruited via flyers posted on the Florida State University campus and in local shops in the Tallahassee area, as well as Craigslist. All prospective participants were pre-screened by phone or email and, if eligible, were given an in-person information session prior to their enrollment. Criteria for inclusion were: overweight or obese men (body mass index (BMI) ≥ 25 kg/m^2^ and body fat > 25%), sedentary (<2 days of planned exercise per week for <45 min per session within the past 6 months), and aged 18–45 years. Exclusion criteria were uncontrolled hypertension (blood pressure > 160/100 mmHg), use of blood pressure or cholesterol medications, diagnosed cardiovascular disease, stroke, diabetes, tobacco users, uncontrolled thyroid or kidney dysfunction or the presence of milk allergies. This study was approved by the Florida State University Human Subjects Committee (HSC#2014.13509). This trial was registered at ClinicalTrials.gov: NCT02211391. Written informed consent and medical history were obtained prior to participation.

### 2.2. Study Design

This crossover study was randomized, double-blind and placebo-controlled. Randomization to the initial condition was conducted using a computerized random number generator [23]. Participants reported to the Institute of Sport Sciences and Medicine at Florida State University on five occasions: once at baseline (morning) and twice (evening and the following morning) during each of the two trial periods. These trial periods were separated by a minimum of 48 h to a maximum of two weeks from one another and the first trial occurred within a week of the baseline visit. All of the morning visits (including baseline), started at 06:00–06:30 h while all of the evening visits started at 18:00–18:30 h. Participants were asked to fast (~8 h) prior to their baseline and morning visits. Participants were also asked to abstain from physical activity, alcohol, and caffeine 24 h prior to each visit.

#### 2.2.1. Morning Visits

##### Anthropometrics and Body Composition

Anthropometric and body composition measurements were assessed only at baseline. Height and weight were measured with a wall-mounted stadiometer (SECA, Hamburg, Germany) and a digital scale (Detecto^®^, Webb City, MO, USA) respectively. Android-gynoid ratio and percent body fat for the whole body and android region were assessed by dual energy X-ray absorptiometry (DXA, Discovery W, Hologic Inc., Bedford, MA, USA) according to manufacturer’s instructions by certified DXA technician.

All subsequent variables listed below were measured during all morning laboratory visits.

##### Appetite

Appetite was assessed with a validated visual analog scale (VAS) [24]. The VAS is 100-mm horizontal scale with opposing extremes of appetite feelings anchored at each end (0 = “not at all” and 100 = “extremely”). Participants rated their subjective feelings of hunger, satiety, and desire to eat, at that moment, by placing a vertical line along the horizontal scale. Ratings were converted to a score in mm and higher scores are indicative of greater feelings of each appetite sensation.

##### Metabolism

Open-circuit indirect calorimetry (ParvoMedics TrueOne 2400 metabolic cart, Sandy, UT, USA) and the ventilated hood method [25] were used to measure oxygen consumption and carbon dioxide production to determine REE and the respiratory exchange ratio (RER). Fat and carbohydrate oxidation rates were calculated from oxygen consumption and RER assuming negligible nitrogen excretion [26]. While assuming negligible nitrogen excretion is a widely accepted method [27,28], it should be noted that this may result in inaccuracies of over-estimation for fat and carbohydrate oxidation. Gas exchange was measured continuously for 45 min and the last 30 min were used for data analysis.

##### Blood Sampling and Analysis

Fasting blood samples were collected via finger prick, as well as from the antecubital vein. Two blood samples were collected via finger prick and subsequently analyzed for glucose using a portable glucose meter (OneTouch Ultramini^®^, LifeScan, Inc., Milpitas, CA, USA) and test strips (OneTouch Ultra^®^, LifeScan, Inc., Wayne, PA, USA). Antecubital samples were collected into non-treated (for insulin and growth hormone analyses) or pre-chilled EDTA-containing (for ghrelin analyses) vacutainers. Pre-chilled EDTA-containing plasma vacutainers were preloaded with 4-(2-aminoethyl)-benzene sulfonylfluoride (AEBSF, Enzo Life Sciences, Farmingdale, NY, USA) prior to sample collection (4 mM final concentration). All samples were centrifuged and serum (300 μL) or plasma (1 mL) aliquots were transferred into microtubes. Serum aliquots were immediately stored at −80 °C for later batch analysis for insulin and growth hormone. Plasma samples were acidified with 200 μL 1N Hydrochloric acid per mL and then stored at −80 °C for ghrelin analysis using previously described methods [29,30]. Concentrations of insulin (IBL-America, Minneapolis, MN, USA) and growth hormone (R & D Systems Inc., Minneapolis, MN, USA) were determined in a single assay using commercially available ELISA kits according to manufacturer’s instructions. The intra-assay coefficient of variation for insulin and growth hormone were 9.6% and 9.5%, respectively. The homeostatic model assessment of insulin resistance (HOMA-IR) was calculated from Equation (1):

HOMA-IR = (fasting insulin (μU/mL) × fasting glucose (mmol/L))/22.5
(1)

Values greater than 2.5 are indicative of insulin resistance [31]. The average of duplicate samples was used for data analysis.

#### 2.2.2. Evening Visits

##### Microdialysis

**Figure 1:**
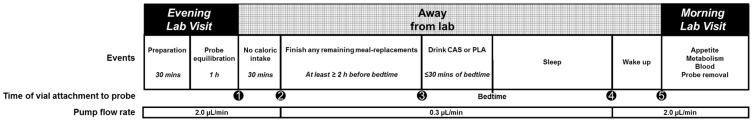
Overview of the trial period. CAS, casein; PLA, placebo. Each sample collection marker (•) indicates the dialysate vial number and the time when the vials were attached to the exit end of each probe (i.e., vial 1 was worn immediately post-equilibration for 30 min). Dialysate samples of interest were the overnight and immediate next morning samples (markers 3 and 4).

The microdialysis technique was used to measure SCAAT lipolysis by continuously monitoring interstitial glycerol concentrations. An overview of the trial period is presented in Figure 1. Participants were instructed to avoid consuming any meal-replacement beverages (described in Section 2.2.3) at least one hour prior to their scheduled evening laboratory visit. Upon arrival to the laboratory for their evening visit participants rested in a semi-recumbent position while researchers sterilized an area ~10 cm from the umbilicus (on the side not typically slept on). Thereafter, the sterilized insertion site was numbed with a topical ethyl chloride spray (Gebauer’s ethyl chloride^®^, Gebauer Company, Cleveland, OH, USA), and two sterilized microdialysis probes (CMA 20 Elite, 10 mm membrane length, 20-kDa cutoff, Harvard Apparatus, Inc., Holliston, MA, USA) were inserted into the SCAAT (5 cm apart). Following probe insertion, a 60-min equilibration period commenced to allow for the initial disturbance from probe insertion to subside. Each probe was attached to separate portable microdialysis pumps (CMA 107, M Dialysis AB, Stockholm, Sweden) that were continuously perfused at 2.0 μL/min or 0.3 μL/min with a solution (perfusate) containing 0.9% sodium chloride (Baxter Healthcare Corporation, Deerfield, IL, USA) and approximately 10 mM ethanol (290 μL of 100% ethanol injected into a 500 mL sodium chloride IV bag). The pumped perfusate was collected in vials at the exit end of each probe (dialysate) according to the sample collection schedule outlined in Figure 1. Each dialysate sample collection shown in Figure 1 represents the time when the vials were attached to the exit end of each probe. The five vials were worn consecutively and for the following durations: (1) worn immediately post-equilibration for 30 min; (2) worn 30 min post-equilibration until ≤30 min before bed; (3) worn ≤30 min before bed until waking (overnight); (4) worn upon waking until arrival at the laboratory (next morning); and (5) worn upon arrival to laboratory until the cessation of their morning visit in which the probes were removed (~1 h after arrival). The dialysate samples of interest were the overnight and immediate next morning samples (sample Collections 3 and 4).

During the equilibration period, participants were provided with verbal and written instructions for the remainder of the evening and into the next morning with regard to changing their sample collection vials, adjusting perfusion flow rates and consuming their nighttime supplement. Immediately prior to leaving the laboratory, the first sample collection vial was attached to collect dialysate outflow (Figure 1). Upon leaving the laboratory (while the first vial was attached), participants were allowed to go through their normal evening activities, which were self-reported to include watching TV, completing school work, and other sedentary behaviors. They were also instructed not to consume any remaining meal-replacement beverages until 30 min after their departure from the laboratory. Thereafter, participants were instructed to consume any remaining meal-replacement beverages at least two hours before they went to bed to ensure that it would digested prior to them consuming their nighttime supplement. While away from the laboratory, participants were asked to store all collection vials in the refrigerator (storage container provided). The following morning (between 06:00 and 06:30 h), participants returned to the laboratory with their dialysate samples on ice. Samples were stored at 4 °C until subsequent (within 24 h) analysis for ethanol to determine blood flow [32]. Thereafter, samples were stored at −80 °C for later batch analysis of dialysate glycerol and glucose according to manufacturer’s instructions using an automated microdialysis analyzer (CMA 600 analyzer, CMA Microdialysis, Solna, Sweden). The average of duplicate measures was used in the calculation of interstitial concentrations from dialysate concentrations.

##### Calculation for Interstitial Glycerol and Glucose

The concentration of glycerol and glucose in the dialysate sample is only representative of a fraction of the actual interstitial concentration, except when perfused at low rates (close to 0 μL/min) as the relative recovery of a substance is ~100% at lower flow rates [33]. In the present study, the zero flow method was used to determine in vivo glycerol and glucose recovery. The zero flow method is based on the assumption that at zero perfusate flow the interstitial concentration of a substance is equal to the dialysate concentration because they are in equilibrium [34,35]. Briefly, the estimated interstitial concentrations were calculated by first plotting log transformed dialysate concentrations measured at 0.3 and 2.0 μL/min against their respective perfusion rates. Linear regression analysis was used to extrapolate the dialysate concentration to that at zero perfusion flow (0 μL/min), which corresponds to the true interstitial concentration. The in vivo recovery rate for each probe was determined as the ratio between the dialysate concentrations at a perfusion rate of 0.3 or 2.0 μL/min and the calculated interstitial concentrations at zero flow [36]. Since the PLA was non-caloric and, hence, mimicked fasting, the in vivo recoveries from the placebo trial for glycerol and glucose were used as a reference point in the calculation of estimate interstitial concentrations for both trials. All interstitial concentrations at 0.3 and 2.0 μL/min were calculated by Equation (2):

Dialysate concentration_flow rate(*x*)_/Recovery_in vivo(Placebo)_ (2)

where, *x* is the flow rate used.

##### Blood Flow

The ethanol technique was used to estimate local blood flow in the area surrounding the probes, by including ethanol in the perfusate. During perfusion, ethanol diffuses through the probe membrane and its removal from the local area is related to blood flow in the vicinity of the probe membrane because ethanol is not metabolized in adipose tissue to any significant extent [32,37]. Within 24 h of sample collection, ethanol concentrations were measured in both the perfusate solutions and dialysate samples using a multi-mode microplate reader (SpectraMax^®^ M5, Molecular Devices, Sunnyvale, CA, USA), as previously described [32]. Perfusate samples were collected at the evening visit prior to attaching the pump to the participant and at the next morning visit after detaching from the participant. Average perfusate concentrations were used in the analysis. The fluorescent NADH product is directly proportional to ethanol in the sample. The ethanol outflow/inflow ratio is calculated according to Equation (3):

Ethanol_dialysate_/Ethanol_perfusate_ (3)

is inversely related to blood flow surrounding the probe [32].

#### 2.2.3. Dietary Logs and Standardization

Participants were asked to maintain their normal dietary and sedentary activity habits during their involvement in the study. Habitual dietary intake was measured with three-day (two weekdays and one weekend day) dietary logs and all dietary log data were analyzed by the same research personnel using the United States Department of Agriculture Super Tracker [38]. Dietary intake was controlled for the entire day on the first day of each trial period (i.e., throughout the day on their scheduled evening visit). Specifically, participants were provided with a choice of chocolate and vanilla flavored meal-replacement beverages (Ensure Plus^®^, 350 kcals, 57% carbohydrate, 28% fat, 15% protein, Abbott Laboratories, Columbus, OH, USA) to match their total daily caloric needs. Caloric needs were calculated from the baseline REE, estimated caloric cost of daily activities for sedentary adults (REE × 0.2), and estimated thermic effect of food (REE × 0.1) used previously in this population (Equation (4)) [39].

Total daily caloric needs = REE + REE × 0.2 + REE × 0.1
(4)

Participants were instructed to consume the meal-replacement beverages at their typical daily meal intervals throughout the day. With the exception of water and their nighttime supplement, no other nutritional sources were ingested. In this way, we were able to assess the impact of CAS under well-controlled dietary intake. Compliance was verified by collecting empty meal-replacement beverage bottles and through verbal questioning.

##### Nighttime Supplementation

The nighttime supplement beverages were chocolate flavored and specifically formulated by Dymatize^®^ Nutrition for use in this study. In a randomized order, participants consumed CAS (total protein content: 30 g, 120 kcals; 25 g micellar protein, 1 g l-Tryptophan, 1 g l-Leucine, 100 mg l-Theanine, 1 g colostrum fraction (bovine)), or a flavor and sensory-matched PLA (0 kcals; non-nutritive sweeteners (sucralose), and stabilizers (gums)). Pre-mixed beverages in a dark-colored shaker bottle were provided to participants immediately prior to leaving the laboratory following the evening visits. Participants were instructed to consume the beverages at least two hours after their last meal-replacement beverage but within 30 min of going to bed (Figure 1).

#### 2.2.4. Sleep Quantity and Quality Assessment

Sleep quantity and quality data were obtained using wrist actigraphy (Fatigue Science Readiband™, Blaine, WA, USA). The actigraphy watch was worn on the non-dominant arm at baseline (~24 h; given at baseline visit and returned the next day) and during both trial periods (~18:00–06:00 h, given at the evening visit and worn until the end of the morning visit the next day). The outcome variables were minutes resting (lying down but not sleeping), minutes sleeping, time it takes to fall asleep (sleep latency), and sleep efficiency (a measure of sleep quality; percentage of time in bed actually sleeping) [40,41]. Sleep efficiency of ≥85% is considered normal [42].

#### 2.2.5. Statistical Analyses

An a priori power analysis was performed from previously published research with interstitial glycerol as the primary outcome variable. A need for 12 participants per group was established to achieve an effect size of 0.80, α = 0.05, with a standard deviation = 16 μmol/L, and difference to detect of 20 μmol/L (treatment-control) (JMP PRO 11, SAS, Cary, NC, USA) [36]. A one-way analysis of variance (ANOVA) was used to identify differences among baseline, CAS and PLA trial for the following variables: REE, RER, all blood markers, appetite, and sleep data. Where significant differences were identified, a Tukey HSD post-hoc analysis was used to locate differences. A Student’s *t*-test was used to compare dietary intake (habitual vs. controlled) and CAS vs. PLA for dialysate glycerol, dialysate glucose and blood flow. Data were analyzed using JMP PRO 11 (SAS, Cary, NC, USA) with significance set at *p* < 0.05. Values are mean ± standard error of the mean (SEM).

## 3. Results

### 3.1. Descriptive Characteristics

During the recruitment period, 103 individuals expressed interest in study participation. Of these individuals, 32 returned their screening document for further review and attended scheduled information sessions. Following the information sessions, 12 individuals expressed interest in enrolling in the study. Descriptive data obtained at baseline are presented in Table 1 and dietary characteristics are in Table 2. One participant did not return his dietary log. Energy intake from the meal-replacement beverages was also included in this table and the difference in caloric intake (habitual (dietary log) vs. controlled (estimated from REE) was 306 kcals with the latter being higher (*p* = 0.16). However, carbohydrate intake was significantly higher during the controlled diet (*p* = 0.00). More importantly, the protein intake (g/kg/day) was not significantly different between the habitual and controlled diets (*p* = 0.90).

### 3.2. Appetite

There were no significant differences in subjective measures of hunger (baseline: 35 ± 6, CAS: 49 ± 6, PLA: 47 ± 6 mm, *p* = 0.21) or satiety (baseline: 36 ± 6, CAS: 36 ± 4, PLA: 39 ± 5 mm, *p* = 0.89). A significant group effect for desire to eat was observed (*p* = 0.03) and post hoc analysis revealed differences between baseline and CAS favoring a greater desire to eat with CAS (baseline: 39 ± 6, CAS: 62 ± 8, PLA: 55 ± 5 mm, *p* = 0.03).

### 3.3. Metabolism

There were no significant differences in REE (baseline: 2150 ± 119, CAS: 2126 ± 111, PLA: 2145 ± 106 kcals/day, *p* = 0.99) or RER (baseline: 0.75 ± 0.02, CAS: 0.76 ± 0.01, PLA: 0.76 ± 0.01, *p* = 0.77). Similarly, neither estimated carbohydrate oxidation (baseline: 0.06 ± 0.03, CAS: 0.08 ± 0.02, PLA: 0.08 ± 0.02 g/min, *p* = 0.76) nor estimated fat oxidation (baseline: 0.13 ± 0.01, CAS: 0.13 ± 0.01, PLA: 0.13 ± 0.01 g/min, *p* = 0.79) were different at any collection time point.

### 3.4. Blood Markers

Blood markers data are presented in Table 3. Glucose measurements during the PLA trial were not available for one participant, so his data were excluded from all glucose and HOMA-IR analyses. There were no differences in glucose, insulin, HOMA-IR, or ghrelin among baseline, CAS and PLA. Participants met the criteria for hyperinsulinemia (fasting insulin > 180 pmol/L) [43] and insulin resistance (HOMA-IR value > 2.5) [31]. Growth hormone data were missing for four participants at various time points due to technical issues (i.e., sample concentrations were unable to be determined for some data points), therefore, only participants with data at all three time points were included in the analysis (*n* = 8). There were no differences in growth hormone at any time point.

### 3.5. Sleep Quantity and Quality

Sleep data at baseline and during the trial periods were only available from a subset of participants (*n* = 5) due to availability of the actigraph watches. Average minutes resting (lying down but not sleeping) were 408.2 ± 26.9 (~6 h, 48 min), 371.0 ± 19.1 (6 h, 12 min), and 311.2 ± 42.1 (5 h, 12 min) (*p* = 0.12) at baseline and during the CAS and PLA, respectively. Average minutes sleeping were 288.0 ± 33.1 (~4 h, 48 min), 261.0 ± 27.4 (4 h, 21 min) and 235.0 ± 42.0 (3 h, 55 min) (*p* = 0.57), for baseline, CAS, and PLA, respectively. Average minutes of sleep latency (the time it takes to fall asleep while resting) were 50.0 ± 22.6, 64.2 ± 25.0, and 17.6 ± 5.2 (*p* = 0.27), for baseline, CAS, and PLA, respectively. The quality of sleep was assessed by the sleep efficiency scores which were 69.9% ± 4.3%, 71.1% ± 8.1% and 74.7% ± 5.6% (*p* = 0.85), for baseline, CAS, and PLA, respectively.

### 3.6. SCAAT Interstitial Glycerol and Glucose

**Figure 2:**
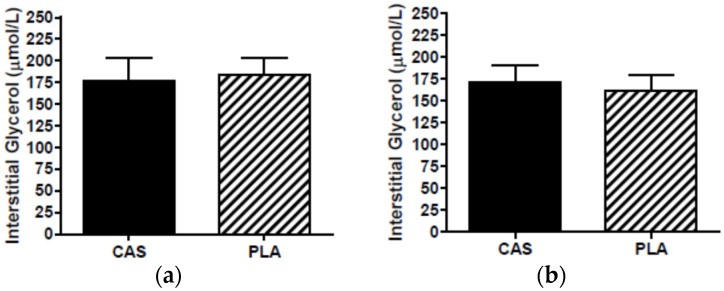
Interstitial glycerol (**a**) overnight during sleep and (**b**) the morning after consumption of casein (CAS) or placebo (PLA); *n* = 11, Mean ± SEM.

**Figure 3:**
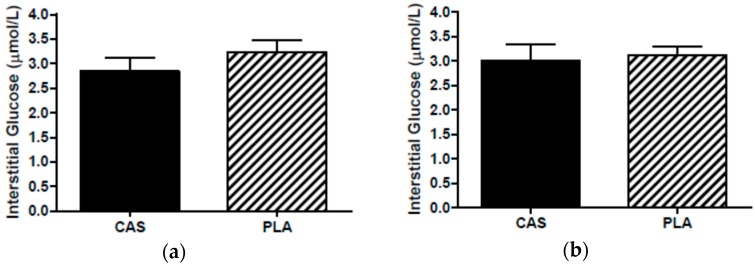
Interstitial glucose (**a**) overnight during sleep and (**b**) the morning after consumption of casein (CAS) or placebo (PLA); *n* = 11, Mean ± SEM.

During the PLA trial, one participant’s morning dialysate collection vial did not have a dialysate sample (from either probe) so this participant’s interstitial data were not included in data analysis as the calculation for interstitial concentrations required both the overnight and next morning PLA samples. The in vivo probe recoveries for PLA were 88% at 0.3 μL/min and 46% at 2.0 μL/min for glycerol and 86% at 0.3 μL/min and 38% at 2.0 μL/min for glucose. The second probe served as a backup in the event that a sample was not collected in the first probe. Interstitial concentrations for one participant’s overnight samples were obtained from the backup probe due to technical difficulties during sleep. Interstitial concentrations were not statistically different between PLA probes (overnight: glycerol, *p* = 0.49; glucose, *p* = 0.81; next morning: glycerol, *p* = 0.30; glucose, *p* = 0.51) and, therefore, the use of data from the backup probe did not affect the results. Overnight (CAS, 177.4 ± 26.7; PLA, 183.8 ± 20.2 μmol/L; *p* = 0.83) and next morning (CAS, 171.6 ± 19.1; PLA, 161.5 ± 18.6 μmol/L, *p* = 0.44) SCAAT interstitial glycerol concentrations were not significantly different between CAS and PLA (Figure 2). Similarly, there were no differences in overnight glucose (CAS, 2.9 ± 0.3; PLA, 3.2 ± 0.2 μmol/L, *p* = 0.16) or next morning glucose (CAS, 3.0 ± 0.3; PLA, 3.1 ± 0.12 μmol/L, *p* = 0.39) between trials (Figure 3).

### 3.7. Blood Flow

Blood flow surrounding the probe is inversely related to the ethanol outflow/inflow ratio. Overnight (CAS, 0.13 ± 0.07; PLA, 0.17 ± 0.07 μmol/L at 0.3μL/min perfusate flow rate; *p* = 0.72) and next morning (CAS, 0.73 ± 0.07; PLA, 0.72 ± 0.07 μmol/L at 2.0 μL/min perfusate flow rate; *p* = 0.96) outflow/inflow ratios were not significantly different between CAS and PLA.

## 4. Discussion

The present study is the first to examine the effect of acute pre-sleep CAS consumption on markers of fat metabolism (lipolysis, substrate utilization, and growth hormone), REE, insulin, glucose, and appetite (subjective and ghrelin) compared to PLA in obese men. We used microdialysis which, for the first time, allowed us to investigate the mechanisms that may alter metabolism and body composition with pre-sleep CAS ingestion. The primary findings were that SCAAT lipolysis, fat oxidation, growth hormone, insulin and glucose were not significantly different between CAS and PLA (hypotheses supported). In addition, CAS did not result in greater REE or suppression of appetite and ghrelin compared to PLA (hypothesis rejected).

Previous acute studies in lean men [9] and obese women [8] have examined next morning substrate utilization and REE as a result of pre-sleep macronutrient intake the night prior. In lean men, bedtime ingestion of calorically-matched macronutrients (CAS, whey protein, carbohydrates) raised REE to a greater extent than the non-nutritive PLA, and, importantly, fat oxidation was not different between CAS and PLA [9]. In obese women, isocaloric CAS, whey protein, and carbohydrate elevated REE, although not significantly and a true non-nutritive PLA was not used thereby limiting the interpretation of these data [8]. Given that REE is the largest component of daily energy expenditure (60%–75%), and that reduced energy expenditure has been linked to weight gain [44], small increments in metabolism through pre-sleep protein intake may be clinically relevant for obese populations. Likewise, if CAS does not blunt lipolysis and, thus, lipolytic rate is not different from a non-nutritive PLA during sleep, then pre-sleep feeding strategies can be developed without concern for effects on lipolysis.

We have previously compared pre-sleep ingestion of calorically matched macronutrients to PLA in lean men and reported that morning fat oxidation was significantly greater for PLA compared to carbohydrates and whey protein but PLA was not different from CAS [9]. Potential mechanisms contributing to the protein-related differences in fat oxidation relative to PLA may be due to alterations in postprandial metabolism during sleep. For example, insulin inhibits fat oxidation, and lower insulin responses have been observed following daytime CAS ingestion compared to whey protein [45]. Insulin is also a potent inhibitor of lipolysis [46] while growth hormone regulates nocturnal lipolysis [47]. In the present study, we hypothesized that pre-sleep CAS consumption would not significantly alter insulin or growth hormone to attenuate SCAAT lipolysis during sleep (measured in a single overnight sample) more than PLA. Thus, lipolytic rate would likely not differ with CAS compared to PLA during sleep, which is consistent with our findings. It is important to mention that protein intake was adequate (above the recommended dietary allowance of 0.8 g/kg of body weight/day) and not significantly different between participants’ self-reported habitual diet and the controlled diet consumed prior to bedtime CAS ingestion (Table 2). Additionally total caloric intake was not different between these diets which allowed us to assess the impact of CAS under well-controlled dietary conditions. No other studies have examined the overnight lipolytic effects of CAS when consumed 30 min prior to sleep. However, our data suggest that postprandial responses following ingestion of low-calorie CAS before sleep may not augment insulin or alter growth hormone concentrations to an appreciable level that would result in lipolytic inhibition compared to the fasted state in obese men. Thus, the additional calories from CAS before sleep did not inhibit lipolysis more than PLA (i.e., fasting) during sleep in obese men and, therefore, likely would not have affected fat oxidation.

Although our findings suggest that CAS prior to sleep does not influence fat metabolism and REE in obese men, we cannot ignore the influence of circulating hormones. We have previously reported increased insulin and insulin resistance in obese women the morning following acute nighttime protein ingestion [8]. However, prior food intake was not well controlled and it is possible that the habitual diet during the day may have confounded the negative effects on insulin. In the present study, morning insulin concentrations did not differ among CAS and PLA trials when food intake was standardized by providing meal-replacement beverages to match the participants total daily caloric needs, however hyperinsulinemia (fasting insulin > 180 pmol/L) [43] and insulin resistance [31] were apparent at all collection periods (Table 3). Under conditions of normal insulin signaling, high levels of insulin would favor fat storage by increasing lipoprotein lipase activity and decreasing hormone-sensitive lipase activity, thereby promoting triglycerides synthesis [46]. Plasma fatty acids and glycerol were not measured in the present study (markers of whole-body lipolysis). However, we observed low RER values (~0.76 ± 0.01) and a corresponding predominance of estimated fat oxidation (fat oxidation: ~0.13 ± 0.01 g/min vs. carbohydrate oxidation: 0.08 ± 0.02 g/min) following both CAS and PLA. While the substrate oxidation rates were estimated from RER it must be noted that protein was not accounted for in these calculations and it is possible that these data may be an over-estimation of true values. Nevertheless, this suggests that plasma free fatty acids and glycerol were elevated, as RER has been shown to be inversely related to plasma fatty acid concentrations [48]. However, it is noteworthy that the addition of a CAS beverage prior to sleep did not further augment insulin concentrations.

It is also possible that the hyperinsulinemia and low RER may be a result of sleep restriction [49]. Rao et al. demonstrated that sleep restriction (~4 h) for five nights leads to lower RER (increase fat utilization) with no change in RMR but increases insulin resistance, cortisol and catecholamines [49]. Despite poor sleep efficiency scores (i.e., below 85%) [42] at baseline and during CAS and PLA trials, no differences in the quality or quantity of sleep between trials were present in the subset of participants (*n* = 5). These data demonstrate that neither microdialysis nor bedtime nutrient ingestion affect sleep quality patterns. Although participants were instructed to consume CAS or PLA within 30 min of going to bed, the time spent lying down but not sleeping was ~6 h, while the average time spent sleeping was ~4.5 h (baseline: 4 h and 48 min ± 33 min; CAS: 4 h and 21 min ± 27 min; PLA: 3 h and 55 min ± 42 min). Thus, it is evident that the participants may have been sleep-restricted at baseline and during the CAS and PLA trials resulting in the low RER values and insulin resistance observed. The present study did not screen for irregular sleep patterns. Hence, future nighttime feeding studies in obese individuals should assess sleep quality and/or screen for sleep disturbances (i.e., sleep apnea).

While other studies have shown that nutrient intake prior to sleep may influence next morning appetite [8,12], whether this holds true in obese men is unknown. We have previously reported that acute pre-sleep ingestion of calorically-matched protein or carbohydrate supplements increased feelings of satiety while concomitantly reducing the desire to eat the following morning, regardless of macronutrient type in obese women [8]. Likewise, less hunger was reported the following morning in response to CAS compared to PLA administration during sleep in elderly men [12]. Surprisingly, our hypothesis that CAS would result in greater appetite suppression compared to PLA was not supported. In fact, our participants reported greater desire to eat during the CAS trial relative to baseline but ghrelin, an orexigenic hormone, did not mediate these changes. Despite previous work showing blunted ghrelin concentrations in obesity [50], obese individuals are highly sensitive to ghrelin’s orexigenic effects as low dose infusion (1 pmol/kg/min) reportedly increases food intake [51]. Changes in subjective appetite, however, were only apparent with a high dose infusion (5 pmol/kg/min) [51]. In the present study, we did not standardize dietary intake prior to the baseline visit, however participants were asked to fast for at least 8 h before their baseline visit. In contrast, liquid meal replacement beverages (Ensure Plus^®^) were used to standardized dietary intake on the days participants consumed CAS and PLA at bedtime (i.e., the day prior to appetite measurements). It is conceivable that the increased feelings for a “desire to eat” may have been mistaken for a “desire to chew” given that their food intake on those days were in liquid form (different from food intake the day prior to baseline) and their subjective feelings of hunger and ghrelin levels were not altered by either trial. While standardizing meal intake with solid meals as opposed to liquid meal replacements would have been ideal, economically were unable to do this in the present study. Regardless, since food intake was standardized and no differences between CAS and PLA trials were present we feel that the same effect would have been observed. Contrary to the present study, data from our laboratory [8,10] and others [12,14,45] have demonstrated a satiating effect of proteins.

## 5. Conclusions 

In conclusion, pre-sleep consumption of 30 g of CAS did not blunt overnight lipolysis compared to a non-nutritive PLA in obese, sedentary, men. The findings in the present study, however, should be interpreted with caution and in the confines of the participant characteristics and study protocol used. However, data exist to support increased daily protein intake for overall health [52] and previous studies demonstrate muscular and overall health benefits to pre-sleep protein ingestion [8,9,10,11,12,13]. Consuming CAS before sleep may be a useful strategy to optimize protein consumption and alter the macronutrient profile in obese, insulin resistant men with no negative acute ramifications on fat metabolism. Future research investigating different types of pre-sleep meals (i.e., liquid vs. solid) and feeding patterns in non-insulin resistant populations are warranted.

## Figures and Tables

**Table 1:** Descriptive characteristics.

	*n* = 12
Age (years)	27.0 ± 2.2
Height (cm)	176.0 ± 2.0
Weight (kg)	112.0 ± 6.6
BMI (kg/m^2^)	36.1 ± 1.9
Body Fat (%)	36.7 ± 1.8
Android Fat (%)	43.8 ± 1.9
A/G ratio	1.19 ± 0.03
Fat Mass (kg)	41.4 ± 4.3
Lean Mass (kg)	66.0 ± 2.7

Mean ± SEM; BMI, body mass index; A/G ratio, android-gynoid ratio.

**Table 2:** Dietary characteristics.

	Habitual	Controlled
Food Intake (kcals)	2489 ± 191 ^^^	2795 ± 154
Carbohydrate		
%	43.8 ± 2.0 ^^^	57.0
grams	274.2 ± 25.9	398.2 ± 22.0 *
Protein		
%	16.8 ± 1.1 ^^^	15.0
grams	103.2 ± 9.3	104.8 ± 5.8
grams/kg/day	0.97 ± 0.1	0.95 ± 0.1
Fat		
%	39.2 ± 2.2 ^^^	28.0
grams	107.7 ± 10.2	86.9 ± 16.6

Habitual dietary intake was measured with three-day dietary logs. Meal replacement beverages (Ensure Plus^®^, provided 350 kcals with 57% carbohydrate, 28% fat, 15% protein per serving) were used for the controlled diet. ^^^, *n* = 11 as one participant did not return their 3-day dietary log. * *p* = 0.001 compared to habitual diet.

**Table 3:** Morning blood markers.

	Baseline	CAS	PLA
Insulin (pmol/L)	202.2 ± 38.4	197.4 ± 37.2	189.6 ± 29.4
Glucose (mmol/L) ^^^	5.0 ± 0.2	4.9 ± 0.2	5.0 ± 0.2
HOMA-IR ^^^	7.9 ± 1.9	7.3 ± 1.6	7.1 ± 1.3
Growth Hormone (μg/L) ^#^	0.16 ± 0.08	0.15 ± 0.07	0.11 ± 0.03
Acyl Ghrelin (pmol/L)	184.7 ± 53.3	183.0 ± 33.7	206.6 ± 51.2
Des Ghrelin (pmol/L)	148.0 ± 30.9	132.0 ± 24.5	143.5 ± 31.6

Mean ± SEM; CAS, casein; PLA, placebo; HOMA-IR, homeostatic model assessment of insulin resistance; ^^^, *n* = 11; ^#^, *n* = 8.

## Abbreviations

The following abbreviations are used in this manuscript:

CAS	Casein protein
REE	Resting energy expenditure
PLA	Placebo
SCAAT	Subcutaneous abdominal adipose tissue
DXA	Dual energy X-ray absorptiometry
VAS	Visual analog scale
RER	Respiratory exchange ratio
HOMA-IR	Homeostatic model assessment of insulin resistance
